# Extended steep ramp test normative values for 19–24-year-old healthy active young adults

**DOI:** 10.1007/s00421-019-04255-x

**Published:** 2019-11-08

**Authors:** M. S. Werkman, B. C. Bongers, T. Blatter, T. Takken, H. Wittink

**Affiliations:** 1 Research Group Lifestyle and Health, Research Center Healthy and Sustainable Living, University of Applied Sciences Utrecht, Utrecht, The Netherlands; 2 Department of Physiotherapy, Leiden University Medical Center, Albinusdreef 2, 2333 ZA Leiden, The Netherlands; 3 Department of Nutrition and Human Movement Sciences, School of Nutrition and Translational Research in Metabolism (NUTRIM), Faculty of Health, Medicine and Life Sciences, Maastricht University, Maastricht, The Netherlands; 4 Department of Epidemiology, Care and Public Health Research Institute (CAPHRI), Faculty of Health, Medicine and Life Sciences, Maastricht University, Maastricht, The Netherlands; 5 SOMT University of Physiotherapy, Amersfoort, The Netherlands; 6 University Medical Center Utrecht, Wilhelmina Children’s Hospital, Child Development and Exercise Center, Utrecht, The Netherlands

**Keywords:** Exercise testing, Field test, Cycle ergometry, Aerobic fitness, Cardiorespiratory fitness

## Abstract

**Purpose:**

To extend currently available sex and age-specific normative values in children and adolescents for the peak work rate (WR_peak_) attained at the steep ramp test (SRT) to healthy active young adults.

**Methods:**

Healthy male and female participants aged between 19 and 24 years were recruited. After screening and anthropometric measurements, participants performed a SRT on a cycle ergometer (increments of 25 W/10 s), monitoring and recording SRT-WR_peak_, heart rate (HR), and blood pressure (BP) at rest and directly after peak exercise.

**Results:**

Fifty-seven participants (31 males and 26 females; median age of 21.3 years) volunteered and were tested. Anthropometrics, resting BP and lung function were all within normal ranges. Ninety-three percent of the participants attained a peak HR (HR_peak_) > 80% of predicted (mean HR_peak_ 87 ± 5% of predicted). No differences were found in resting and peak exercise variables between females and males, except for absolute SRT-WR_peak_ (350 W [Q1: 306; Q3: 371] and 487 W [Q1: 450; Q3: 517], respectively) and SRT-WR_peak_ normalized for body mass (relative SRT-WR_peak_; 5.4 ± 0.5 and 6.2 ± 0.6 W/kg, respectively). Low-to-moderate correlations (*ρ* [0.02–0.71]) were observed between SRT-WR_peak_ and anthropometric variables for females and males separately. Extended reference curves (8–24-year-old subjects) for SRT performance show different trends between male and female subjects when modelled against age, body height, and body mass.

**Conclusions:**

The present study provides sex-, age-, body height-, and body mass-related normative values (presented as reference centiles) for absolute and relative SRT performance throughout childhood and early adulthood.

**Electronic supplementary material:**

The online version of this article (10.1007/s00421-019-04255-x) contains supplementary material, which is available to authorized users.

## Introduction

Cardiopulmonary exercise testing (CPET) is a procedure involving an incremental, graded exercise test of 8–12 min in duration that provides essential diagnostic, prognostic, and evaluative information in a broad spectrum of (chronic) diseases (Vanhees et al. 2005). Traditionally, results from CPET are widely considered to be the criterion standard for measurement of cardiorespiratory fitness (CRF), reflected by the oxygen uptake at peak exercise (*V*O_2peak_) (Weisman et al. 2003).

Despite its clinical relevance, the use of formal CPET seems to be underused in current usual clinical practice. It has its (minor) disadvantages in the light of time investment, availability of the necessary equipment, and the required practical and theoretical skills of the laboratory staff (Stevens et al. 2010). Because of these limitations, there is a need for less sophisticated clinical exercise testing procedures to assess CRF that can easily be applied in usual care. Currently there are some alternatives of interest, such as the steep ramp test (SRT), which does not require respiratory gas analysis measurements.

The SRT has originally been developed as a short clinical exercise test to personalize and evaluate interval training in patients with heart failure (Meyer et al. 1996). The SRT is performed on a calibrated cycle ergometer up to volitional maximal exertion, and the attained peak work rate (SRT-WR_peak_) is its primary outcome measure. Given its strong association with *V*O_2peak_, SRT performance has been reported to provide a strong and reliable indication of CRF in different (patient) populations in the cardiopulmonary, metabolic, and oncological domains within a broad age-range (Bongers et al. 2015; Braam et al. 2015; De Backer et al. 2007; Rozenberg et al. 2015; Strookappe et al. 2016; Stuiver et al. 2017). Unfortunately, no such evidence about reliability and validity is available for healthy young adults.

Additionally, in healthy children and adolescents (Bongers et al. 2013a) and in adult cancer survivors (De Backer et al. 2007; Stuiver et al. 2017) and type II diabetes (Rozenberg et al. 2015) the SRT has recently been found to be a reliable and valid exercise test to predict *V*O_2peak_ from the attained SRT-WR_peak_ and anthropometric variables. However, these equations lack precision and induce large prediction margins for *V*O_2peak_, which hampers their clinical usability for individuals (Stuiver et al. 2017). Moreover, predicting *V*O_2peak_ from SRT performance is simply not necessary when adequate normative values for its primary outcome measure (SRT-WR_peak_) are available for a broad age-range.

Unfortunately, such normative values are only available for 8–19-year-old children and adolescents (Bongers et al. 2013b). Adequate normative values are of evident importance to track CRF in the young adult general health setting as it is for young adult patients with (chronic) diseases in which exercise testing and exercise training are part of usual care. Therefore, this study aimed to extend the currently available sex- and age-specific normative values for SRT performance in 19–24- year-old healthy active young adults.

## Methods

### Design

This study is part of the STEep Ramp test Norm values Utrecht Maastricht (STERNUM) study, which has been approved by the Medical Ethical Committee of the University Medical Center Utrecht (16–675/M). To extend the currently available normative values (Bongers et al. 2013b), this part of the study has been designed as an observational (cross-sectional) study in which self-reported healthy active young adults participated. The range 19–24 years was chosen based on clinical considerations to provide extended norms for the same exercise test for follow-up of CRF during transition from the pediatric to the adult health care setting male and female participants without contra-indications (see “Measurements”) for maximal exercise testing in the age between 19 and 24 years were recruited from the Utrecht University Campus region, dominantly at the Department of Movement Studies, University of Applied Sciences, Utrecht, The Netherlands. Informed consent was obtained from all individual participants included in the study. Participants were recruited and tested between March 2017 and November 2018. For statistical purposes, it was aimed to have a larger number of participants of 19 and 24 years of age.

All participants provided written informed consent and all procedures performed in studies involving human participants were in accordance with the ethical standards of the institutional and/or national research committee (Medical Ethical Committee of the University Medical Center Utrecht) and with the 1964 Helsinki Declaration and its later amendments or comparable ethical standards. The data sets generated during and/or analyzed during the current study are available from the corresponding author on reasonable request.

### Measurements

Participants were recruited using flyers and defined themselves as healthy and compliant with the Dutch Physical Activity Guidelines (Weggemans et al. 2018). Before testing procedures, participants were screened for eligibility by administrating the American College of Sports Medicine (ACSM) preparticipation screening questionnaire, including questions about medical history (e.g., presence of known cardiovascular, pulmonary, neurological, or musculoskeletal disease), exercise-related complaints, and cardiovascular risk factors (Balady et al. 1998). Additionally, an anamnesis, blood cholesterol level measurement, resting supine electrocardiogram, and resting supine blood pressure were taken to confirm the outcome of the preparticipation questionnaire. Furthermore, pulmonary function was tested to confirm statements in the questionnaire, as well as to exclude the possibility of a ventilatory constraint-limiting exercise performance. Contra-indications for maximal exercise testing as described in the American Thoracic Society/American College of Chest Physicians Statement on Cardiopulmonary Exercise Testing were asked for in the anamnesis (ATS/ACCP 2003). “Healthy” was then operationalized as the combination of the following items: (1) self-reported healthy, without complaints during physical activity or exercise, (2) self-reported and anamnesis-based absence of contra-indications for maximal exercise, (3) self-reported non-smoking, and (4) normal pulmonary function and normal blood pressure (BP) and electrocardiogram (ECG) at rest and during exercise. All procedures and tests were performed by two trained and experienced clinical exercise physiologists (TB and MW). When no contraindications were identified, the formal testing procedure continued.

Before exercise testing, each participant’s body height and body mass were measured. Body mass index [BMI (kg/m^2^)] was calculated as the body mass divided by body height squared. Body surface area [BSA (m^2^)] was estimated using the equation of Haycock et al. (1978). Skinfold thickness was measured at the right side triceps, biceps, subscapular, and suprailiacal site with a Harpenden skinfold caliper (Baty International, West Sussex, United Kingdom). Subcutaneous fat as a percentage of body mass was then calculated out of the four-site sum of skinfolds (Durnin and Womersley 1974), where after fat-free mass (FFM) was calculated. Lung function, operationalized as the forced expiratory volume in 1 second (FEV_1_) measured in L and expressed as a percentage of predicted (Quanjer et al. 1993), was measured while sitting upright on the cycle ergometer. Participants performed three maximal flow-volume maneuvers through a face mask (Hans Rudolph Inc., Kansas City, MO, USA) that was connected to a calibrated metabolic cart (MetaLyzer 3BR2, Cortex Biophysik GmbH, Leipzig, Germany) with software (MetaSoft Studio v5.8.5 SR2, Cortex Biophysik GmbH, Leipzig, Germany).

The SRT was performed according to a previously published procedure in adult patients with heart failure (Meyer et al. 1996). Cycling was performed on an electronically braked cycle ergometer (Lode Corival, Lode BV, Groningen, The Netherlands). Heart rate (HR) was monitored continuously using a 12-lead ECG (Custocor Custo Med, Ottobrunn, Germany). Blood pressure (BP) was monitored at rest and directly after exercise cessation using the Riva Rocci method, without duplication or triplication of the measurement due to pragmatic reasons (Maxistabil 3 sphygmo manometer, Heine Adult-cuff, Speidel & Keller, Jungingen, Germany). Each participant was carefully instructed about the testing procedure, including information about the importance to perform a true maximal effort and how to handle in the case of any possible complaints. Participants were already familiar with the SRT procedure as it is part of the standard pallet of exercise tests taught in the first study year. Afterwards, the participant was fitted with all equipment. After assessment of baseline HR and BP during a three-minute resting period while seated on the cycle ergometer, the test started with 2 min of unloaded cycling. Thereafter, work rate was linearly increased by a ramp protocol equaling 25 W each 10 s. Participants were instructed to maintain a pedaling rate between 60 and 80 revolutions per minute. Strong verbal encouragement was given by the investigators until the participant terminated the SRT because of voluntary exhaustion or when their pedaling frequency fell definitely < 60 revolutions per minute, despite strong verbal encouragement. The attained peak exercise values at the SRT (WR_peak_, HR_peak_, and BP_peak_) and time until exhaustion (SRT duration in seconds, excluding the unloaded cycling phase) were recorded and the attainment of a maximal effort was noted in each participant’s case report form. Performance was defined as maximal effort by the test leader when participants demonstrated subjective signs of exhaustion (e.g., unsteady biking, sweating, facial flushing, clear unwillingness to continue despite strong verbal encouragements). HR_peak_ was calculated and presented as percentage of predicted using the Nes formula (Predicted HR_peak_ = $211-(0.64\times \mathrm{a}\mathrm{g}\mathrm{e}))$ (Nes et al. 2013).

### Data analysis

Data analysis concerning anthropometrics, male–female differences and associations between parameters has been focused on the current 19–24-year-old group. Data analysis was performed with the Statistical Package for the Social Sciences (SPSS version 25, IBM SPSS Inc, Chicago, Illinois). All data are expressed as mean ± standard deviation (SD) and [range] or as median [Q1; Q3] when not normally distributed. Normality of the data was tested with the Kolmogorov–Smirnov test. Differences between males and females were examined with independent sample t-tests, or Mann–Whitney *U* test as appropriate. To counteract the possible problem of multiple comparisons the Bonferroni method was performed (0.05/*k* = 16 comparisons). Pearson (*r*) or Spearman (*ρ*) correlation coefficients were calculated to examine associations between the SRT-WR_peak_ and various relevant anthropometric variables. The raw data previously collected in 8- to 19-year-old males and females by Bongers et al. (Bongers et al. 2013b) were combined with the current 19–24-year-old raw data set to develop sex-, age-, body height-, and body mass-specific normative values for 8–24-year-old males and females. Reference centiles were developed for absolute SRT-WR_peak_ (in W) and body mass- and FFM-corrected “relative” SRT-WR_peak_ (in W/kg and W/kg FFM, respectively) and variables were plotted against age, body height, and body mass. The Lambda-Mu-Sigma (LMS) Chart Maker Pro version 2.3 software (The Institute of Child Health, London, UK) was used, which fits smooth centile curves to reference data (Cole and Green 1992). According to our statistical expert there is no formal possibility to perform a power calculation for setting up norm values using the LMS method. Similar to the previous Bongers study (Bongers et al. 2013b), participants were stratified in 1-year clusters stratified on sex and age. Obviously, in that study a larger (*n* = 252), but also a population wider in age range (8–19) was included. As such, growth and maturation did play a much more dominant role in the development of reference curves in that pediatric and adolescent group than expected in the current young adult group. Therefore, our sample of 70 healthy young adults seems to be a feasible and sufficient number to construct generalizable norm values. A *p* value < 0.05 was considered statistically significant.

## Results

Fifty-seven participants (31 males and 26 females; median age 21.3 years [Q1: 20.1; Q3: 23.4 years], range 19.1–24.9 years) volunteered to participate in the study and were eligible to perform exercise testing after preparticipation screening. Of these, nine participants (16%) had a right bundle branch block at the resting electrocardiogram, which was interpreted as no contraindication for maximal exercise testing by the consulting cardiologist. BMI, resting BP and lung function were all within normal ranges. As expected, males had higher values for body height, body mass, BSA, FFM, and absolute FEV_1_. Additionally, all participants were apparently healthy, non-smoking, and physically active [compliant to the Dutch Physical Activity Guidelines (Weggemans et al. 2018)] based on self-reported data and the administration of the ACSM screening questionnaire.

Most anthropometric, resting, and exercise variables, except age, body height, BSA, FEV_1_, resting systolic BP, and SRT-WR_peak_, were normally distributed.

Participant characteristics and resting hemodynamic variables are presented in Tables 1 and 2. During and after exercise testing, only one (1.8%) adverse event occurred (near syncope after maximal exercise), which recovered after several minutes in supine position. There were no events necessitating full 12-lead ECG control afterwards. All participants performed a maximal effort during the SRT, based on subjective signs of exhaustion or until pedal frequency fell definitely < 60 rpm, despite strong verbal encouragement. Peak exercise variables are presented in Table 2. Ninety-three percent of the participants attained a HR_peak_ > 80% of predicted, with a mean HR_peak_ of 87 ± 5% of predicted.

**Table 1:** Participant characteristics

	Females Mean ± SD [range]/median [Q1; Q3]	Males Mean ± SD [range]/median [Q1; Q3]	*p* (95% CI)*
Age (years)	21.0 [19.8; 22.5]	22.7 [20.7; 23.8]	0.02
Body height (cm)	169.3 [164.9; 177.4]	186.0 [183.6; 189.0]	< 0.01**
Body mass (kg)	63.7 ± 7.3 [49.0–81.0]	77.0 ± 9.1 [47.0–99.0]	< 0.01 [8.8 to 17.7]**
BMI (kg/m^2^)	22.0 ± 1.9 [18.8–26.8]	22.2 ± 2.1 [16.2–25.9]	0.74 [− 0.9 to 1.3]
BSA	1.7 [1.7; 1.8]	2.0 [1.9; 2.1]	< 0.01**
FFM (kg)	47.1 ± 5.7 [37.4–57.8]	68.4 ± 7.8 [44.4–80.0]	< 0.01**
FEV_1_ (L)	3.77 [3.42; 3.90]	5.17 [4.80; 5.66]	< 0.01**
FEV_1_ (%pred)	104 ± 11 [86–128]	104 ± 10 [82–121]	0.91 [− 6 to 5]

*BMI* body mass index, *CI* confidence interval, *FEV*_*1*_ forced expiratory volume in one second, *FFM* fat-free mass, *Q1* 25th percentile, *Q3* 75th percentile, *%pred* presented as percentage of predicted

*95%CI only presented when tested parametrically

**Statistically significant after Bonferroni correction (*α* < 0.003 (0.05/*k* = 17)

**Table 2:** Resting and exercise parameters

	Females Mean ± SD [range]/median [Q1; Q3]	Males Mean ± SD [range]/median [Q1; Q3]	*p* (95% CI)*
Rest			
HR (beats/min)	82 ± 13 [55–104]	80 ± 11 [60–103]	0.58 [− 8 to 5]
Systolic BP (mmHg)	113 [108; 128]	118 [111; 132]	0.24
Diastolic BP (mmHg)	72 ± 9 [49–84]	72 ± 10 [41–89]	0.90 [− 5 to 6]
Peak exercise			
HR_peak_ (beats/min)	171 ± 9 [155–188]	173 ± 10 [154–196]	0.66 [− 4 to 6]
HR_peak_ (%pred)	87 ± 5 [79–95]	88 ± 5 [79–99]	0.51 [− 2 to 3]
Systolic BP_peak_ (mm Hg)	165 ± 17 [129–193]	171 ± 20 [134–221]	0.24 [− 4 to 17]
Diastolic BP (mm Hg)	69 ± 9 [49–82]	65 ± 17 [41–105]	0.32 [− 11 to 4]
SRT-WR_peak_ (W)	350 [306; 371]	487 [450; 517]	< 0.01**
SRT-WR_peak_ (W/kg)	5.4 ± 0.5 [4.0–6.2]	6.2 ± 0.6 [4.5–7.6]	< 0.01 [0.5 to 1.1]**
SRT-WR_peak_ (W/kg FFM)	7.3 ± 0.8 [5.4–8.6]	7.0 ± 0.7 [5.6–9.1]	0.12 [− 0.69 to 0.08]
SRT-duration (s)	140 [122; 148]	195 [180; 207]	< 0.01**

*BP* blood pressure, *CI* confidence interval, *FFM* fat-free mass, *HR* heart rate, *HR*_*peak*_ peak heart rate, *Q1* 25th percentile, *Q3* 75th percentile, *s* seconds, *SRT-WR*_*peak*_ peak work rate attained at the steep ramp test, *%pred* presented as percentage of predicted

*95% CI only presented when tested parametrically

**Statistically significant after Bonferroni correction (*α* < 0.003 (0.05/*k* = 18)

No differences were found in resting and peak exercise variables between females and males, except for absolute SRT-WR_peak_ (350 W [Q1: 306; Q3: 371] and 487 W [Q1: 450; Q3: 517], respectively; *p* < 0.01), relative SRT-WR_peak_ (5.4 ± 0.5 and 6.2 ± 0.6 W/kg, respectively; *p* < 0.01) and SRT duration (140 s [Q1: 122; Q3: 148] and 195 s [Q1: 180; Q3: 207], respectively; *p* < 0.01). The hemodynamic response to maximal exercise at the SRT was comparable between females and males as reflected by comparable HR and BP values at peak exercise. Peak exercise variables are presented in Table 2.

Low-to-high correlations (*ρ* [0.02–0.71]) were observed between absolute SRT-WR_peak_ and various anthropometric variables, as shown in Table 3 for females and males separately. As expected, absolute SRT-WR_peak_ was positively and statistically significant associated with body height, body mass, FFM, BSA, and FEV_1_ (*ρ* [0.46–0.71]); all *p* values < 0.017). No significant correlation was observed between age and absolute SRT-WR_peak_ in females and males (*ρ* − 0.33; *p* = 0.10 and *ρ* 0.02; *p* = 0.90, respectively; see Table 3).

**Table 3:** Spearman Rank correlation coefficients for SRT-WR_peak_ and selected anthropometric variables in 19- to 24-year old females and males

Variable	Females		Males	
	*ρ*	*p* value	*ρ*	*p* value
Age (years)	− 0.33	0.10	0.02	0.90
Body mass (kg)	0.54	< 0.01*	0.61	< 0.01*
Body height (cm)	0.46	0.02*	0.55	0.01*
BSA (m^2^)	0.53	0.01*	0.67	< 0.01*
FFM (kg)	0.55	< 0.01*	0.71	< 0.01*
FEV_1_ (L)	0.47	0.02*	0.50	0.01*

*BSA* body surface area, *FEV*_*1*_ forced expiratory volume in one second, *FFM* fat-free mass, *ρ* Spearman rank correlation coefficient

*Statistically significant

**Fig. 1:**
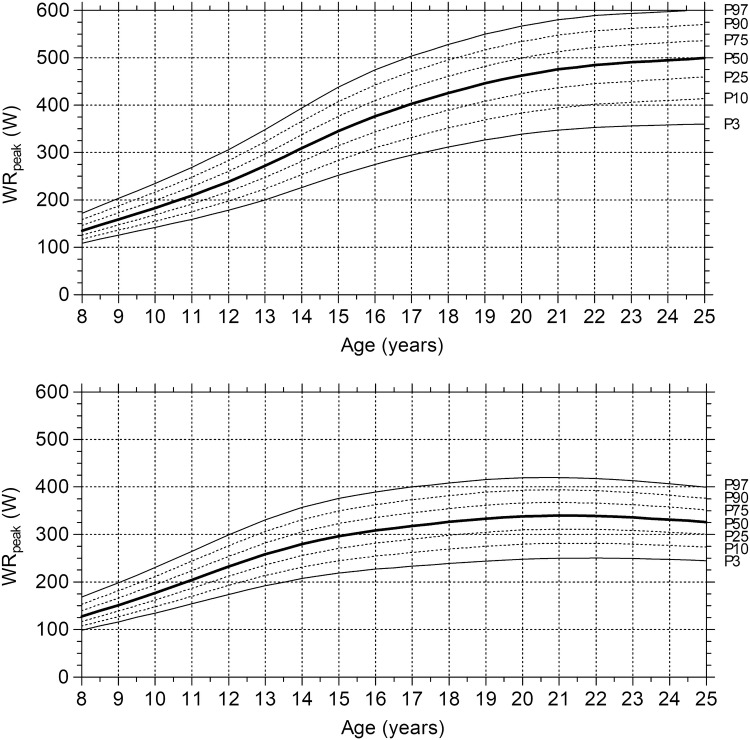
Age-related centile curves for the SRT-WR_peak_ for male (upper charts) and female (lower charts) subjects. Bold solid lines represent the 50th centile (P50); dashed lines correspond to the 10th, 25th, 75th, and 90th centiles (P10, P25, P75, and P90, respectively); and solid lines indicate the 3rd and 97th centiles (P3 and P97, respectively)

Figure 1 shows the age-specific reference centile charts for absolute SRT-WR_peak_ for male subjects (upper chart) and female subjects (lower chart). In male subjects, the values demonstrate an almost linear increase in SRT-WR_peak_ until the age of approximately 15 years. After 15 years, the increase plateaus. In female subjects, the SRT-WR_peak_ increased until the age of 19 years, where after it plateaus.

**Fig. 2:**
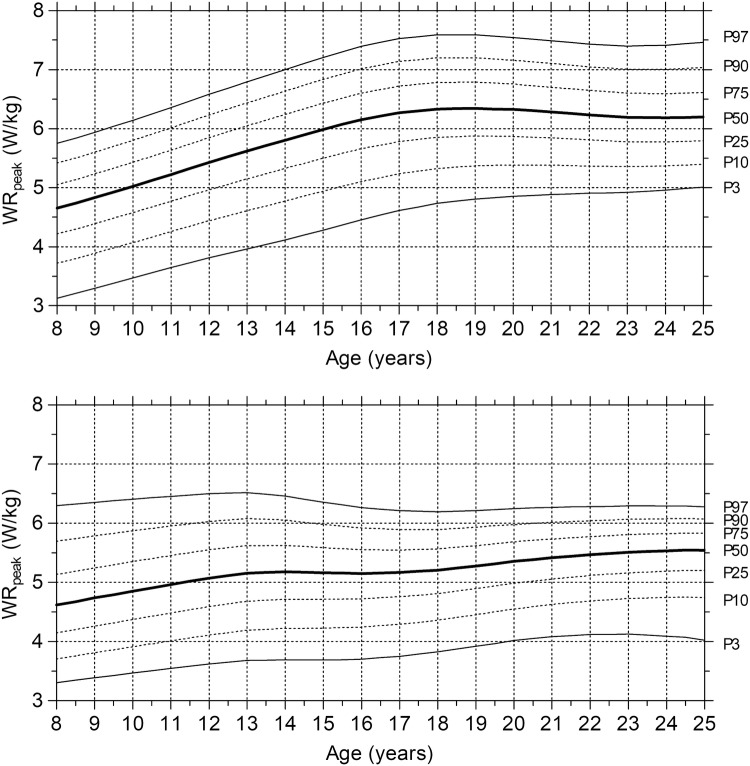
Age-related centile curves for the Relative SRT-WR_peak/kg_ for male (upper charts) and female (lower charts) subjects. Bold solid lines represent the 50th centile (P50); dashed lines correspond to the 10th, 25th, 75th, and 90th centiles (P10, P25, P75, and P90, respectively); and solid lines indicate the 3rd and 97th centiles (P3 and P97, respectively)

When normalized for body mass, Fig. 2 (upper chart) shows an almost linear increase of the relative SRT-WR_peak_ in ages up to 19 years in male subjects, where after it stabilizes. In female subjects (lower chart), relative SRT-WR_peak_ showed only a relatively small increase (+0.6 W/kg) until age 14 years, where after it plateaus until the age of 18 years. After 18 years, SRT-WR_peak_ shows a small increase (+0.3 W/kg) again.

Additional Figures with body height- and body mass-related centile curves for the absolute SRT-WR_peak_ and relative SRT-WR_peak_ are presented as Online Resources. When absolute SRT-WR_peak_ is plotted against body height (Online Resource 1), the upper chart (male subjects) shows an exponential increase in absolute SRT-WR_peak_ until the body height of 155 cm. When taller than 155 cm, absolute SRT-WR_peak_ seems to increase linearly with body height. In female subjects (lower chart), absolute SRT-WR_peak_ seems to increase linearly from the body height of 145 cm. In shorter female subjects, absolute SRT-WR_peak_ seems to increase exponentially. Relative SRT-WR_peak_ as a function of body height (Online Resource 2), shows a wave-form trend for relative SRT-WR_peak_ with increasing body height in male subjects (upper chart). The lower bound of body height shows a slight decrease in relative SRT-WR_peak_ with increasing body height up to 145 cm. Thereafter, relative SRT-WR_peak_ increases exponentially until 175 cm, where after it starts to level off. In female subjects (lower chart), a stable relative SRT-WR_peak_ is observed throughout the entire body height range of 125–195 cm of approximately 5.0 W/kg.

When absolute SRT-WR_peak_ was plotted as a model of body mass (Online Resource 3), the same trends were found for male subjects (upper chart) as in Online Resource 1 for body height. In female subjects, absolute SRT-WR_peak_ increases linearly until a body mass of 60 kg. Thereafter it starts to level off. The same optimum body masses for relative SRT-WR_peak_ are observed when plotted against body mass (Online Resource 4). For both male subjects (upper chart) and female subjects (lower chart), relative peak performance occurred at a body mass of approximately 60 kg. In male subjects a plateau remains until approximately 80 kg, where after relative SRT-WR_peak_ starts to decrease. In female subjects, relative SRT-WR_peak_ directly starts to decrease after 60 kg.

Figures were constructed based on the values in the tables in the Online Resource 5, presenting sex-, age-, body height-, body mass-, and FFM-related normative values for absolute and relative (normalized for body mass and FFM) SRT-WR_peak_ for 8–24-year-old males and females, expressed as centile scores (Online Resource 5).

## Discussion

The purpose of this study was to extend the currently available sex- and age-specific normative values and centile curves for SRT performance in 19–24-year-old healthy young adults. This study provides these extended normative values for the absolute SRT-WR_peak_ and relative SRT-WR_peak_ in 19–24-year-old males and females, and combined them with the existing data of 8–19-year-old boys and girls. This provides a complete overview concerning the development of SRT performance throughout childhood into early adulthood.

As expected, peak work rates increased with age in both males and females. This trend continued in males until the age of 24 years, but leveled off after 19 years in females, which is comparable to the Dubowy study in which children and adults show the same curves for absolute *V*O_2peak_ values attained during an incremental treadmill-based exercise test (Dubowy et al. 2008). It seems that the impact of growth and maturation proceeds in boys and males until 24 years, but becomes less dominant in effect on performance in girls and females after 19 years. This less-dominant impact of growth on SRT performance is confirmed with the lower correlations (*ρ* [0.02–0.71]) of growth-related anthropometric variables in this young adult study population compared to overall strong correlations in the younger (<19 years) population reported by Bongers et al. (2013b). Additionally, SRT performance is suggested to be more prominently limited by the local skeletal muscle anaerobic energy system, which is suggested to contribute much more to the energy supply during the SRT than in the more aerobic considered CPET (Rozenberg et al. 2015) and which is less dependent on growth and maturation. Hence, SRT-WR_peak_ values in both females and males are much higher than what would be expected at the conventional CPET. As there is a strong positive association between ramp slope and peak power output and a strong negative association between time until exhaustion and peak power output (Morton 2011), the high SRT-WR_peak_ is explained by the much shorter time until exhaustion at the SRT (median 159 s in this study) versus the recommended time to induce exhaustion within 10 min for the conventional CPET (ATS/ACCP 2003).

Normalized for body mass, a linear increase in relative SRT performance is seen in male subjects until the age of 18 years, whereas a relatively stable trend is observed in female subjects. Again, comparable curves are noted for body mass-corrected “relative” *V*O_2peak_ values (ml/kg/min) in the Dubowy treadmill protocol; however, the plateau-phase starts later in boys and males at the age of 24 (Dubowy et al. 2008).

The availability of these extended normative values has two main (clinical) advantages. First, given the reported excellent associations of SRT-WR_peak_ with *V*O_2peak_ in previous studies (Bongers et al. 2015; Braam et al. 2015; De Backer et al. 2007; Rozenberg et al. 2015; Strookappe et al. 2016; Stuiver et al. 2017), these extended norms provide the clinician with reference values to follow CRF throughout the early (childhood, adolescence, and young adulthood) life-span of an individual during health and disease. This provides the clinician with a tool to initiate interventions as exercise training in time and only when necessary. Additionally, in pediatric chronic diseases which start to manifest in (early) childhood (e.g., asthma, cystic fibrosis, pediatric cardiology, oncology), these norms can be used to adequately follow the development of patients CRF from childhood into early adulthood using the same exercise test and the same set of norm values. Second, these extended SRT norms provide clinicians with an alternative to track CRF without the necessity for respiratory gas analysis. We advise to base the choice of which reference curves to use on clinical considerations. As the current normative values are presented as body height, body mass, and FFM-specific, reference curves can be used for shorter and taller, as well as for over- and underweight patients.

Additionally, in that way, the SRT can be used as a screening tool for the necessity to perform a standard CPET. As advised by Bongers et al. (2013b), when the absolute SRT-WR_peak_ or relative SRT-WR_peak_ at a validly performed SRT are below the third percentile for age-, body height- or body mass-related curves, a CPET can be used to differentiate between cardiorespiratory, metabolic, or local skeletal muscle origin of lowered CRF. Being the golden standard for assessment of CRF, measurement of *V*O_2peak_ with a CPET should always be the test of choice when used for diagnostic purposes.

Although the SRT is reported to be safe and already part of usual care and regular follow-up in diverse deconditioned patient populations, precautions should be taken to enhance safety during and after testing, especially when performed in the presence of cardiopulmonary disease. Exercise HR_peak_ (mean 87% of predicted HR_peak_; 93% of participants attained > 80% predicted HR_peak_) and mean BP response [mean increase in systolic BP of 49 ± 19 mm Hg (range + 3 to + 109 mm Hg)] were evident, and were comparable with maximal dynamic cycle ergometry values in elite athletes in which exaggerated BP responses are observed (Pressler et al. 2013). Based on this observation and previously reported (close-to) maximal cardiopulmonary responses (~SRT-HR_peak_ > 90% of CPET-HR_peak_) induced by the SRT in clinical and healthy populations (Bongers et al. 2013a; Braam et al. 2015; Chura et al. 2012; De Backer et al. 2010; Meyer et al. 1997; Rozenberg et al. 2015; Werkman et al. 2011), the SRT should be regarded as a maximal exercise test (Holland et al. 2014). We advise to monitor HR and BP at rest, at peak exercise, and throughout at least 5 minutes of active and passive recovery. In patients with pulmonary diseases, pulse oximetry can be used to monitor peripheral oxygenation continuously.

One methodological consideration is the possibility of a small selection bias as the main part of the participants were recruited at the University of Applied Sciences, Department of Movement Studies. Participants may therefore be more physically active and more fit than sex- and age-related peers, as well as possibly having a higher socioeconomic status. However, in another part of the STERNUM study, a representative subgroup also performed a CPET, in which their *V*O_2peak_ was measured as gold standard for CRF. The mean measured *V*O_2peak_ equaled 99% of predicted [range 74–134%]) (*V*O_2peak_ data from Van de Poppe et al. 2019), indicating that the current study participants are an adequate reflection of the general population considering CRF. Furthermore, general evaluation of the student’s background from introduction surveys when subscribing for the Department of Movement Studies reveals that most physical therapy students are the first in the family starting a study in higher education. This suggests that the currently collected normative data are generalizable. However, still, the relatively small sample size contributes to the limitation as it precludes the assumption of a high degree of similarity with the general population which could not be accounted for.

In future, it is of evident clinical necessity to further extend these normative values for healthy elderly to be able to follow CRF in older (chronic) patients were exercise testing is part of usual care. Furthermore, disease-specific norms are necessary.

In conclusion, the present study provides sex-, age-, body height-, body mass-, and FFM-related normative values (presented as reference centiles) for absolute and relative SRT performance throughout childhood and early adulthood. Reference curves for SRT performance show different trends between male and female subjects when modelled against age, body height, and body mass, highlighting the necessity for these normative values to follow CRF easily over time in health and disease.

## Electronic supplementary material

Below is the link to the electronic supplementary material.

Supplementary material 1 (TIF 82,942 kb)

Supplementary material 2 (TIF 84,064 kb)

Supplementary material 3 (TIF 82,714 kb)

Supplementary material 4 (TIF 84,064 kb)

ESM_5.pdf Tables with age-, sex-, body height-, and body mass-related normative values for absolute SRT-WRpeak and relative SRT_WRpeak for 8–24-year-old males and females, expressed as mean ± SD (PDF 605 kb)

## Abbreviations

ACSM: American College of Sports Medicine
BMI: Body mass index
BP: Blood pressure
BSA: Body surface area
COPD: Chronic obstructive pulmonary disease
CPET: Cardiopulmonary exercise testing
CRF: Cardiorespiratory fitness
ECG: Electrocardiogram
FEV_1_: Forced expiratory volume in one second
FFM: Fat-free mass
HR: Heart rate
ICC_2,1_: Two-way random intra class correlation coefficients
LMS: Least-Mean-Square
NA: Not applicable
Q1: 25th percentile
Q3: 75th percentile
RER: Respiratory exchange ratio
RPM: Revolutions per minute
SD: Standard deviation
SDC: Smallest detectable change
SEE: Standard error of the estimate
SEM: Standard error of measurement
SpO_2_: Peripheral oxygen saturation
SPSS: Statistical Package for the Social Sciences
SRT: Steep ramp test
*V*CO_2_: Carbon dioxide production
VE: Minute ventilation
*V*O_2peak_: Oxygen uptake at peak exercise
WR_peak_: Peak work rate
%pred: Presented as percentage of predicted
ΔBP: Systolic BPpeak − systolic BPrest
ΔHR: HRpeak − HRrest
