# Supplementary material for: Extended steep ramp test normative values for 19–24-year-old healthy active young adults
Source: Eur J Appl Physiol. 2019 Nov 8;120(1):107–15. doi: 10.1007/s00421-019-04255-x (PMC6969871; doi:10.1007/s00421-019-04255-x)
Supplement: Supplementary file 5 — ESM_5.pdf Tables with age-, sex-, body height-, and body mass-related normative values for absolute SRT-WRpeak and relative SRT_WRpeak for 8–24-year-old males and females, expressed as mean ± SD (PDF 605 kb) [file 421_2019_4255_MOESM5_ESM.pdf]

Supplemental Digital Content 1

Supplementary Table 1. Age- and sex-related reference centiles for absolute SRT-WR_{peak}.

Age	SRT-WR_{peak} (W)													
	Males							Females						
(years)	P3	P10	P25	**P50**	P75	P90	P97	P3	P10	P25	**P50**	P75	P90	P97
8.0	109	117	126	**135**	146	158	172	99	107	117	**128**	140	153	168
8.5	118	127	136	**147**	159	173	188	108	117	128	**140**	153	167	183
9.0	126	136	147	**159**	173	187	204	116	127	139	**151**	166	181	198
9.5	134	145	158	**171**	186	202	219	126	137	150	**164**	180	196	214
10.0	142	155	168	**183**	199	216	235	135	148	162	**177**	194	211	231
10.5	151	165	180	**196**	213	232	252	144	159	174	**191**	209	227	248
11.0	159	175	191	**209**	228	248	269	154	170	187	**205**	224	244	265
11.5	169	186	204	**224**	244	265	288	164	181	199	**219**	239	260	282
12.0	178	197	217	**238**	260	283	306	174	193	212	**233**	254	276	299
12.5	189	210	232	**255**	278	303	327	183	203	224	**246**	268	291	315
13.0	200	223	247	**272**	297	322	348	192	214	236	**259**	282	306	331
13.5	213	238	264	**290**	317	344	371	200	222	246	**269**	294	319	344
14.0	226	253	281	**309**	337	366	394	208	231	256	**280**	306	331	357
14.5	239	268	298	**327**	357	386	416	213	238	263	**289**	314	340	367
15.0	252	283	314	**345**	376	407	438	219	245	271	**297**	323	350	376
15.5	264	296	329	**361**	393	425	456	223	250	276	**303**	329	356	383
16.0	275	310	343	**377**	410	442	475	227	254	282	**309**	336	363	390
16.5	285	321	356	**390**	423	457	489	230	258	286	**313**	341	368	395
17.0	295	332	368	**403**	437	471	504	234	262	290	**318**	346	373	400
17.5	303	342	379	**414**	449	483	516	236	266	294	**322**	350	377	404
18.0	312	352	389	**426**	461	495	528	239	269	298	**327**	354	382	409
18.5	319	360	399	**436**	471	506	539	242	272	302	**330**	358	385	412
19.0	327	369	408	**446**	482	516	550	244	275	305	**334**	362	389	416
19.5	333	376	416	**455**	491	525	558	246	277	307	**336**	364	391	418
20.0	339	383	424	**463**	499	534	567	248	280	310	**338**	366	393	420
20.5	343	389	431	**469**	506	541	574	249	281	310	**339**	367	394	420
21.0	348	394	437	**476**	513	548	581	250	281	311	**340**	368	394	420
21.5	350	398	441	**480**	517	552	585	250	281	311	**340**	367	393	419
22.0	353	402	445	**485**	522	557	589	251	281	311	**339**	366	393	418
22.5	355	404	448	**488**	525	559	592	250	281	310	**338**	364	390	416
23.0	356	406	450	**491**	527	562	594	250	280	308	**336**	363	388	413
23.5	357	408	453	**493**	530	564	596	249	278	307	**334**	360	386	410
24.0	358	410	455	**495**	532	566	598	248	277	305	**332**	358	383	407
24.5	359	412	457	**497**	534	568	600	247	275	303	**329**	355	379	403
25.0	360	414	459	**500**	536	570	602	245	274	301	**326**	352	376	400

Abbreviation: SRT-WR_{peak}=peak work rate attained at the Steep Ramp Test

Supplementary Table 2. Age- and sex-related reference centiles for SRT-WR_{peak} normalized for body mass.

Age (years)	SRT-WR_{peak} (W/kg) Males P3	P10	P25	**P50**	P75	P90	P97	Females P3	P10	P25	**P50**	P75	P90	P97
8.0	3.1	3.7	4.2	**4.7**	5.1	5.4	5.8	3.3	3.7	4.1	**4.6**	5.1	5.7	6.3
8.5	3.2	3.8	4.3	**4.7**	5.1	5.5	5.8	3.3	3.8	4.2	**4.7**	5.2	5.7	6.3
9.0	3.3	3.9	4.4	**4.8**	5.2	5.6	5.9	3.4	3.8	4.3	**4.7**	5.2	5.8	6.4
9.5	3.4	4.0	4.5	**4.9**	5.3	5.7	6.0	3.4	3.9	4.3	**4.8**	5.3	5.8	6.4
10.0	3.5	4.1	4.6	**5.0**	5.4	5.8	6.1	3.5	3.9	4.4	**4.9**	5.4	5.9	6.4
10.5	3.6	4.2	4.7	**5.1**	5.5	5.9	6.3	3.5	4.0	4.4	**4.9**	5.4	5.9	6.4
11.0	3.6	4.3	4.8	**5.2**	5.6	6.0	6.4	3.5	4.0	4.5	**5.0**	5.5	6.0	6.5
11.5	3.7	4.3	4.9	**5.3**	5.7	6.1	6.5	3.6	4.1	4.5	**5.0**	5.5	6.0	6.5
12.0	3.8	4.4	5.0	**5.4**	5.8	6.2	6.6	3.6	4.1	4.6	**5.1**	5.6	6.0	6.5
12.5	3.9	4.5	5.1	**5.5**	5.9	6.3	6.7	3.7	4.1	4.6	**5.1**	5.6	6.1	6.5
13.0	4.0	4.6	5.1	**5.6**	6.0	6.4	6.8	3.7	4.2	4.7	**5.2**	5.6	6.1	6.5
13.5	4.0	4.7	5.2	**5.7**	6.1	6.5	6.9	3.7	4.2	4.7	**5.2**	5.6	6.1	6.5
14.0	4.1	4.8	5.3	**5.8**	6.2	6.6	7.0	3.7	4.2	4.7	**5.2**	5.6	6.1	6.5
14.5	4.2	4.9	5.4	**5.9**	6.3	6.7	7.1	3.7	4.2	4.7	**5.2**	5.6	6.0	6.4
15.0	4.3	4.9	5.5	**6.0**	6.4	6.8	7.2	3.7	4.2	4.7	**5.2**	5.6	6.0	6.4
15.5	4.4	5.0	5.6	**6.1**	6.5	6.9	7.3	3.7	4.2	4.7	**5.2**	5.6	5.9	6.3
16.0	4.5	5.1	5.7	**6.2**	6.6	7.0	7.4	3.7	4.2	4.7	**5.2**	5.6	5.9	6.3
16.5	4.5	5.2	5.7	**6.2**	6.7	7.1	7.5	3.7	4.3	4.7	**5.2**	5.5	5.9	6.2
17.0	4.6	5.2	5.8	**6.3**	6.7	7.1	7.5	3.8	4.3	4.8	**5.2**	5.5	5.9	6.2
17.5	4.7	5.3	5.8	**6.3**	6.8	7.2	7.6	3.8	4.3	4.8	**5.2**	5.6	5.9	6.2
18.0	4.7	5.3	5.9	**6.3**	6.8	7.2	7.6	3.8	4.4	4.8	**5.2**	5.6	5.9	6.2
18.5	4.8	5.3	5.9	**6.3**	6.8	7.2	7.6	3.9	4.4	4.9	**5.2**	5.6	5.9	6.2
19.0	4.8	5.4	5.9	**6.3**	6.8	7.2	7.6	3.9	4.5	4.9	**5.3**	5.6	5.9	6.2
19.5	4.8	5.4	5.9	**6.3**	6.8	7.2	7.6	4.0	4.5	4.9	**5.3**	5.7	6.0	6.2
20.0	4.9	5.4	5.9	**6.3**	6.8	7.2	7.5	4.0	4.5	5.0	**5.4**	5.7	6.0	6.2
20.5	4.9	5.4	5.9	**6.3**	6.7	7.1	7.5	4.1	4.6	5.0	**5.4**	5.7	6.0	6.3
21.0	4.9	5.4	5.8	**6.3**	6.7	7.1	7.5	4.1	4.6	5.1	**5.4**	5.7	6.0	6.3
21.5	4.9	5.4	5.8	**6.3**	6.7	7.1	7.5	4.1	4.7	5.1	**5.4**	5.8	6.0	6.3
22.0	4.9	5.4	5.8	**6.2**	6.6	7.0	7.4	4.1	4.7	5.1	**5.5**	5.8	6.0	6.3
22.5	4.9	5.4	5.8	**6.2**	6.6	7.0	7.4	4.1	4.7	5.1	**5.5**	5.8	6.1	6.3
23.0	4.9	5.4	5.8	**6.2**	6.6	7.0	7.4	4.1	4.7	5.2	**5.5**	5.8	6.1	6.3
23.5	4.9	5.4	5.8	**6.2**	6.6	7.0	7.4	4.1	4.7	5.2	**5.5**	5.8	6.1	6.3
24.0	5.0	5.4	5.8	**6.2**	6.6	7.0	7.4	4.1	4.7	5.2	**5.5**	5.8	6.1	6.3
24.5	5.0	5.4	5.8	**6.2**	6.6	7.0	7.4	4.1	4.8	5.2	**5.5**	5.8	6.1	6.3
25.0	5.0	5.4	5.8	**6.2**	6.6	7.0	7.5	4.0	4.7	5.2	**5.5**	5.8	6.1	6.3

Abbreviation: SRT-WR_{peak}=peak work rate attained at the Steep Ramp Test

Supplementary Table 3. Age- and sex-related reference centiles for SRT-WR_{peak} normalized for fat-free mass.

Age (years)	SRT-WR_{peak} (W/kg FFM) Males P3	P10	P25	**P50**	P75	P90	P97	Females P3	P10	P25	**P50**	P75	P90	P97
8.0	4.3	4.9	5.3	**5.7**	6.0	6.3	6.6	4.8	5.1	5.5	**5.9**	6.4	6.9	7.4
8.5	4.4	5.0	5.4	**5.8**	6.1	6.4	6.7	4.8	5.2	5.6	**6.0**	6.5	6.9	7.5
9.0	4.5	5.1	5.5	**5.9**	6.2	6.5	6.8	4.9	5.2	5.6	**6.1**	6.5	7.0	7.5
9.5	4.6	5.2	5.6	**6.0**	6.4	6.7	7.0	4.9	5.3	5.7	**6.1**	6.6	7.1	7.6
10.0	4.8	5.3	5.7	**6.1**	6.5	6.8	7.1	5.0	5.4	5.8	**6.2**	6.7	7.1	7.6
10.5	4.9	5.4	5.8	**6.3**	6.6	7.0	7.3	5.0	5.4	5.8	**6.3**	6.7	7.2	7.7
11.0	5.0	5.5	6.0	**6.4**	6.7	7.1	7.4	5.0	5.5	5.9	**6.4**	6.8	7.3	7.7
11.5	5.1	5.6	6.1	**6.5**	6.9	7.2	7.6	5.1	5.5	6.0	**6.4**	6.9	7.3	7.8
12.0	5.2	5.7	6.2	**6.6**	7.0	7.4	7.7	5.1	5.6	6.0	**6.5**	7.0	7.4	7.9
12.5	5.2	5.8	6.2	**6.7**	7.1	7.5	7.8	5.1	5.6	6.1	**6.5**	7.0	7.5	7.9
13.0	5.3	5.9	6.3	**6.8**	7.2	7.6	8.0	5.1	5.6	6.1	**6.6**	7.1	7.5	8.0
13.5	5.4	5.9	6.4	**6.9**	7.3	7.7	8.1	5.1	5.7	6.2	**6.6**	7.1	7.6	8.0
14.0	5.5	6.0	6.5	**7.0**	7.4	7.8	8.2	5.2	5.7	6.2	**6.7**	7.1	7.6	8.0
14.5	5.6	6.1	6.6	**7.0**	7.5	7.9	8.4	5.2	5.7	6.2	**6.7**	7.2	7.6	8.0
15.0	5.7	6.2	6.6	**7.1**	7.6	8.0	8.5	5.2	5.7	6.2	**6.7**	7.2	7.6	8.1
15.5	5.7	6.2	6.7	**7.2**	7.7	8.1	8.6	5.2	5.7	6.2	**6.7**	7.2	7.6	8.1
16.0	5.8	6.3	6.8	**7.2**	7.7	8.2	8.7	5.2	5.7	6.3	**6.7**	7.2	7.7	8.1
16.5	5.9	6.3	6.8	**7.3**	7.8	8.3	8.8	5.2	5.8	6.3	**6.8**	7.2	7.7	8.1
17.0	5.9	6.4	6.8	**7.3**	7.8	8.3	8.8	5.2	5.8	6.3	**6.8**	7.3	7.7	8.1
17.5	6.0	6.4	6.9	**7.3**	7.8	8.3	8.9	5.3	5.8	6.4	**6.9**	7.3	7.8	8.2
18.0	6.0	6.4	6.9	**7.3**	7.8	8.4	8.9	5.3	5.9	6.4	**6.9**	7.4	7.8	8.3
18.5	6.0	6.4	6.9	**7.3**	7.8	8.4	8.9	5.4	5.9	6.5	**7.0**	7.4	7.9	8.3
19.0	6.0	6.4	6.8	**7.3**	7.8	8.3	8.9	5.5	6.0	6.5	**7.0**	7.5	7.9	8.4
19.5	6.0	6.4	6.8	**7.3**	7.8	8.3	8.9	5.5	6.1	6.6	**7.1**	7.6	8.0	8.4
20.0	6.0	6.4	6.8	**7.3**	7.8	8.3	8.9	5.6	6.1	6.7	**7.2**	7.6	8.1	8.5
20.5	6.0	6.4	6.8	**7.2**	7.7	8.3	8.8	5.6	6.2	6.7	**7.2**	7.7	8.1	8.6
21.0	6.0	6.3	6.7	**7.2**	7.7	8.2	8.8	5.7	6.2	6.8	**7.3**	7.7	8.2	8.6
21.5	5.9	6.3	6.7	**7.1**	7.6	8.2	8.8	5.7	6.3	6.8	**7.3**	7.8	8.2	8.7
22.0	5.9	6.3	6.7	**7.1**	7.6	8.1	8.8	5.7	6.3	6.9	**7.4**	7.8	8.3	8.7
22.5	5.9	6.2	6.6	**7.1**	7.6	8.1	8.7	5.7	6.3	6.9	**7.4**	7.9	8.3	8.7
23.0	5.9	6.2	6.6	**7.0**	7.5	8.1	8.7	5.7	6.4	6.9	**7.4**	7.9	8.3	8.8
23.5	5.8	6.2	6.5	**7.0**	7.5	8.0	8.7	5.7	6.4	7.0	**7.5**	7.9	8.4	8.8
24.0	5.8	6.1	6.5	**6.9**	7.4	8.0	8.7	5.7	6.4	7.0	**7.5**	8.0	8.4	8.8
24.5	5.8	6.1	6.5	**6.9**	7.4	8.0	8.7	5.7	6.4	7.0	**7.5**	8.0	8.4	8.8
25.0	5.8	6.1	6.4	**6.8**	7.3	7.9	8.7	5.7	6.4	7.0	**7.6**	8.0	8.5	8.8

Abbreviations: FFM=fat-free mass; SRT-WR_{peak}=peak work rate attained at the Steep Ramp Test

Supplementary Table 4. Body height- and sex-related reference centiles for absolute SRT-WR_{peak}.

Body height	SRT-WR_{peak} (W)													
	Males							Females						
(cm)	P3	P10	P25	**P50**	P75	P90	P97	P3	P10	P25	**P50**	P75	P90	P97
125	124	128	131	**134**	137	140	142	110	116	121	**126**	130	134	138
127	125	130	134	**138**	142	145	148	110	117	122	**128**	132	137	141
129	126	132	137	**142**	146	150	153	110	117	124	**130**	135	140	145
131	127	134	141	**146**	151	155	159	111	119	126	**133**	139	144	150
133	128	136	144	**150**	155	160	165	113	121	129	**137**	143	149	155
135	129	139	147	**154**	161	166	171	115	125	133	**141**	149	156	162
137	131	142	151	**159**	166	173	179	118	128	138	**147**	155	163	170
139	133	146	156	**165**	173	180	187	122	133	143	**153**	162	171	179
141	137	150	161	**171**	180	188	196	126	138	150	**160**	171	180	190
143	141	155	167	**178**	188	197	206	132	145	157	**169**	180	191	202
145	147	161	174	**186**	197	207	217	138	152	165	**178**	190	203	215
147	154	168	182	**195**	207	219	230	145	160	174	**188**	202	215	229
149	161	176	191	**205**	219	232	245	153	168	183	**198**	214	229	245
151	170	185	200	**216**	231	246	261	162	178	194	**210**	227	244	261
153	178	194	210	**227**	244	261	278	171	187	204	**222**	240	259	278
155	187	204	221	**239**	257	276	295	181	198	215	**234**	253	274	296
157	196	214	232	**251**	271	291	313	191	208	226	**246**	267	290	314
159	206	224	244	**264**	285	308	331	202	219	238	**258**	280	305	332
161	216	235	256	**277**	300	324	350	212	230	249	**270**	294	320	349
163	227	247	268	**291**	316	342	369	223	240	260	**281**	306	333	364
165	237	259	281	**306**	332	359	389	232	250	270	**292**	317	345	378
167	249	271	295	**320**	348	377	408	241	259	280	**302**	327	356	389
169	260	283	308	**335**	364	395	428	250	268	289	**312**	337	366	398
171	271	296	322	**351**	381	413	447	258	277	298	**321**	346	374	406
173	283	309	336	**366**	397	430	466	267	286	307	**329**	354	382	412
175	294	321	350	**381**	413	448	484	275	294	315	**338**	363	389	419
177	305	334	364	**396**	429	465	502	284	303	324	**347**	371	397	425
179	316	346	378	**411**	446	482	519	293	313	334	**356**	380	405	431
181	327	359	392	**426**	462	499	537	302	322	343	**365**	388	412	438
183	337	371	406	**442**	478	515	554	312	332	353	**374**	397	420	444
185	348	383	420	**457**	494	532	570	322	342	363	**384**	405	428	450
187	357	395	434	**472**	510	548	586	333	352	372	**393**	414	435	457
189	367	407	447	**487**	525	564	602	343	363	382	**402**	422	443	463
191	376	419	461	**501**	541	579	617	353	373	392	**412**	431	450	470
193	385	430	474	**516**	556	595	633	364	383	402	**421**	439	458	476
195	393	442	487	**530**	571	610	648	374	393	412	**430**	448	466	483

Abbreviation: SRT-WR_{peak}=peak work rate attained at the Steep Ramp Test

Supplementary Table 5. Body height- and sex-related reference centiles for SRT-WR_{peak} normalized for body mass.

Body height (cm)	SRT-WR_{peak} (W/kg) Males P3	P10	P25	**P50**	P75	P90	P97	Females P3	P10	P25	**P50**	P75	P90	P97
125	3.3	4.0	4.5	**5.0**	5.5	5.9	6.2	3.3	3.7	4.2	**4.8**	5.4	6.0	6.8
127	3.2	3.9	4.5	**5.0**	5.4	5.8	6.2	3.3	3.7	4.2	**4.7**	5.3	5.9	6.7
129	3.2	3.9	4.5	**4.9**	5.4	5.8	6.1	3.3	3.7	4.2	**4.7**	5.3	5.9	6.6
131	3.2	3.9	4.4	**4.9**	5.3	5.7	6.1	3.3	3.7	4.2	**4.7**	5.2	5.8	6.5
133	3.2	3.9	4.4	**4.9**	5.3	5.7	6.0	3.3	3.7	4.2	**4.7**	5.2	5.8	6.4
135	3.2	3.9	4.4	**4.9**	5.3	5.7	6.0	3.3	3.7	4.2	**4.7**	5.2	5.8	6.4
137	3.3	3.9	4.4	**4.9**	5.3	5.7	6.0	3.3	3.8	4.2	**4.7**	5.2	5.8	6.4
139	3.3	3.9	4.4	**4.9**	5.3	5.7	6.1	3.4	3.8	4.2	**4.7**	5.2	5.8	6.4
141	3.3	3.9	4.5	**5.0**	5.4	5.8	6.1	3.4	3.8	4.3	**4.8**	5.3	5.8	6.4
143	3.4	4.0	4.5	**5.0**	5.4	5.8	6.2	3.4	3.9	4.3	**4.8**	5.3	5.8	6.4
145	3.4	4.1	4.6	**5.1**	5.5	5.9	6.3	3.5	3.9	4.4	**4.8**	5.3	5.9	6.4
147	3.5	4.1	4.7	**5.2**	5.6	6.0	6.4	3.5	4.0	4.4	**4.9**	5.4	5.9	6.4
149	3.6	4.2	4.8	**5.3**	5.7	6.2	6.6	3.6	4.0	4.5	**5.0**	5.4	5.9	6.5
151	3.7	4.3	4.9	**5.4**	5.9	6.3	6.7	3.6	4.1	4.5	**5.0**	5.5	6.0	6.5
153	3.7	4.4	4.9	**5.5**	6.0	6.4	6.9	3.7	4.1	4.6	**5.1**	5.5	6.0	6.5
155	3.8	4.5	5.0	**5.6**	6.1	6.5	7.0	3.7	4.2	4.6	**5.1**	5.6	6.0	6.5
157	3.9	4.5	5.1	**5.6**	6.2	6.6	7.1	3.7	4.2	4.7	**5.2**	5.6	6.0	6.5
159	3.9	4.6	5.2	**5.7**	6.2	6.7	7.2	3.7	4.2	4.7	**5.2**	5.6	6.1	6.5
161	4.0	4.6	5.2	**5.8**	6.3	6.8	7.3	3.8	4.3	4.8	**5.2**	5.7	6.1	6.5
163	4.0	4.7	5.3	**5.8**	6.4	6.9	7.3	3.8	4.3	4.8	**5.3**	5.7	6.1	6.4
165	4.1	4.7	5.3	**5.9**	6.4	6.9	7.4	3.8	4.3	4.8	**5.3**	5.7	6.1	6.4
167	4.1	4.8	5.4	**5.9**	6.5	7.0	7.4	3.8	4.4	4.9	**5.3**	5.7	6.0	6.4
169	4.2	4.8	5.4	**6.0**	6.5	7.0	7.4	3.7	4.4	4.9	**5.3**	5.7	6.0	6.3
171	4.2	4.9	5.5	**6.0**	6.5	7.0	7.5	3.7	4.3	4.8	**5.3**	5.6	5.9	6.2
173	4.3	4.9	5.5	**6.1**	6.6	7.0	7.5	3.7	4.3	4.8	**5.2**	5.6	5.9	6.2
175	4.3	5.0	5.6	**6.1**	6.6	7.0	7.4	3.6	4.3	4.8	**5.2**	5.5	5.8	6.1
177	4.4	5.0	5.6	**6.1**	6.6	7.0	7.4	3.6	4.3	4.8	**5.2**	5.5	5.8	6.0
179	4.4	5.1	5.6	**6.1**	6.6	7.0	7.4	3.5	4.3	4.8	**5.2**	5.5	5.7	6.0
181	4.5	5.1	5.6	**6.1**	6.6	7.0	7.4	3.4	4.3	4.8	**5.1**	5.4	5.7	5.9
183	4.6	5.2	5.7	**6.2**	6.6	7.0	7.3	3.4	4.3	4.7	**5.1**	5.4	5.6	5.8
185	4.6	5.2	5.7	**6.2**	6.6	7.0	7.3	3.3	4.2	4.7	**5.1**	5.3	5.6	5.8
187	4.7	5.3	5.8	**6.2**	6.6	6.9	7.3	3.2	4.2	4.7	**5.0**	5.3	5.5	5.7
189	4.8	5.3	5.8	**6.2**	6.6	6.9	7.2	3.1	4.2	4.6	**5.0**	5.2	5.4	5.6
191	4.9	5.4	5.8	**6.2**	6.5	6.9	7.2	3.0	4.1	4.6	**4.9**	5.1	5.3	5.5
193	5.0	5.4	5.8	**6.2**	6.5	6.8	7.1	2.9	4.1	4.6	**4.9**	5.1	5.3	5.4
195	5.1	5.5	5.8	**6.2**	6.5	6.7	7.0	2.8	4.1	4.5	**4.8**	5.0	5.2	5.3

Abbreviation: SRT-WR_{peak}=peak work rate attained at the Steep Ramp Test

Supplementary Table 6. Body mass- and sex-related reference centiles for absolute SRT-WR_{peak}.

Body mass (kg)	SRT-WR_{peak} (W)													
	Males							Females						
	P3	P10	P25	**P50**	P75	P90	P97	P3	P10	P25	**P50**	P75	P90	P97
24	88	101	113	**124**	134	144	153	89	102	115	**128**	141	154	167
26	94	109	122	**134**	145	156	165	96	109	123	**137**	150	164	178
28	101	117	132	**145**	157	168	178	102	116	131	**145**	160	174	189
30	107	126	141	**155**	168	180	191	108	124	139	**155**	170	185	200
32	114	134	151	**167**	181	193	205	115	132	148	**164**	180	196	211
34	122	143	162	**179**	194	208	220	122	140	157	**174**	190	207	223
36	129	153	174	**192**	208	223	236	129	148	166	**184**	201	218	235
38	138	164	186	**206**	223	239	254	137	157	176	**195**	213	230	247
40	146	175	200	**220**	239	256	272	145	166	186	**206**	224	242	260
42	156	187	213	**236**	256	274	291	153	176	197	**217**	236	254	272
44	165	199	227	**251**	272	292	310	162	185	207	**228**	247	266	285
46	174	211	240	**266**	289	309	328	170	195	217	**239**	259	278	297
48	184	222	254	**281**	304	326	346	179	204	228	**250**	271	290	309
50	193	234	267	**295**	320	343	364	187	214	238	**261**	282	303	322
52	203	246	280	**310**	336	360	382	196	224	249	**272**	294	315	334
54	214	258	293	**324**	352	377	400	205	234	259	**283**	305	326	346
56	225	270	306	**338**	367	393	417	214	243	269	**294**	316	338	358
58	237	282	319	**352**	382	409	434	222	252	279	**304**	327	348	369
60	249	294	332	**366**	396	424	451	229	260	288	**313**	336	358	379
62	260	305	344	**379**	410	439	466	235	267	295	**321**	345	367	388
64	270	316	356	**391**	424	454	482	241	273	302	**328**	353	375	396
66	280	327	367	**403**	437	467	496	245	279	308	**335**	359	382	403
68	290	337	378	**415**	449	481	510	249	283	313	**340**	365	387	409
70	299	347	389	**427**	461	494	524	252	287	318	**345**	369	392	413
72	308	357	399	**438**	473	506	537	255	291	321	**349**	373	396	417
74	316	366	409	**449**	485	518	549	258	294	325	**352**	376	399	420
76	323	374	419	**459**	495	529	561	260	297	328	**355**	379	401	422
78	329	382	427	**468**	505	539	571	263	299	330	**357**	381	404	424
80	333	388	435	**477**	514	549	581	266	302	333	**360**	384	405	426
82	337	394	442	**484**	522	557	589	269	305	336	**362**	386	407	427
84	339	399	448	**491**	529	564	596	272	308	338	**365**	388	409	429
86	340	403	453	**497**	535	570	602	276	311	341	**367**	390	411	431
88	340	406	458	**502**	541	575	607	279	315	344	**370**	393	413	432
90	339	409	462	**507**	546	580	612	283	318	348	**373**	395	416	434
92	337	411	466	**511**	550	584	616	288	322	351	**376**	398	418	436
94	334	412	469	**515**	554	588	619	292	326	355	**379**	401	421	439
96	329	414	473	**519**	558	592	623	296	330	358	**382**	404	423	441
98	322	415	476	**523**	562	596	626	301	334	362	**386**	407	426	443
100	313	415	478	**526**	566	599	629	306	339	366	**389**	410	428	445

Abbreviation: SRT-WR_{peak}=peak work rate attained at the Steep Ramp Test

Supplementary Table 7. Body mass- and sex-related reference centiles for SRT-WR_{peak} normalized for body mass.

Body mass (kg)	SRT-WR_{peak} (W/kg) Males P3	P10	P25	**P50**	P75	P90	P97	Females P3	P10	P25	**P50**	P75	P90	P97
24	3.8	4.3	4.7	**5.1**	5.5	5.8	6.2	3.6	4.1	4.7	**5.2**	5.8	6.3	6.8
26	3.8	4.3	4.7	**5.1**	5.5	5.9	6.3	3.6	4.1	4.7	**5.2**	5.7	6.2	6.8
28	3.8	4.3	4.7	**5.2**	5.6	5.9	6.3	3.6	4.1	4.6	**5.2**	5.7	6.2	6.7
30	3.8	4.3	4.7	**5.2**	5.6	6.0	6.4	3.6	4.1	4.6	**5.2**	5.7	6.2	6.7
32	3.8	4.3	4.8	**5.2**	5.7	6.1	6.4	3.6	4.1	4.6	**5.1**	5.6	6.1	6.6
34	3.7	4.3	4.8	**5.3**	5.7	6.1	6.5	3.6	4.1	4.6	**5.1**	5.6	6.1	6.6
36	3.7	4.3	4.9	**5.3**	5.8	6.2	6.6	3.6	4.1	4.6	**5.1**	5.6	6.1	6.5
38	3.7	4.4	4.9	**5.4**	5.9	6.3	6.7	3.6	4.1	4.6	**5.1**	5.6	6.1	6.5
40	3.8	4.4	5.0	**5.5**	6.0	6.4	6.8	3.6	4.2	4.7	**5.2**	5.6	6.1	6.5
42	3.8	4.5	5.1	**5.6**	6.1	6.5	7.0	3.6	4.2	4.7	**5.2**	5.6	6.1	6.5
44	3.8	4.5	5.1	**5.7**	6.2	6.6	7.1	3.7	4.2	4.7	**5.2**	5.6	6.0	6.5
46	3.8	4.6	5.2	**5.7**	6.3	6.7	7.2	3.7	4.2	4.7	**5.2**	5.6	6.0	6.4
48	3.9	4.6	5.2	**5.8**	6.3	6.8	7.3	3.7	4.3	4.7	**5.2**	5.6	6.0	6.4
50	3.9	4.7	5.3	**5.9**	6.4	6.9	7.3	3.7	4.3	4.8	**5.2**	5.6	6.0	6.4
52	3.9	4.7	5.4	**5.9**	6.5	6.9	7.4	3.8	4.3	4.8	**5.2**	5.6	6.0	6.4
54	4.0	4.8	5.4	**6.0**	6.5	7.0	7.4	3.8	4.3	4.8	**5.2**	5.7	6.0	6.4
56	4.0	4.8	5.4	**6.0**	6.5	7.0	7.5	3.8	4.3	4.8	**5.2**	5.7	6.0	6.4
58	4.1	4.8	5.5	**6.1**	6.6	7.1	7.5	3.8	4.4	4.8	**5.2**	5.6	6.0	6.4
60	4.2	4.9	5.5	**6.1**	6.6	7.1	7.5	3.8	4.4	4.8	**5.2**	5.6	6.0	6.3
62	4.2	4.9	5.5	**6.1**	6.6	7.1	7.5	3.8	4.3	4.8	**5.2**	5.6	5.9	6.3
64	4.2	5.0	5.6	**6.1**	6.6	7.1	7.5	3.8	4.3	4.7	**5.2**	5.5	5.9	6.2
66	4.3	5.0	5.6	**6.1**	6.6	7.1	7.5	3.7	4.2	4.7	**5.1**	5.5	5.8	6.1
68	4.3	5.0	5.6	**6.1**	6.6	7.1	7.5	3.7	4.2	4.6	**5.0**	5.4	5.7	6.0
70	4.3	5.0	5.6	**6.1**	6.6	7.0	7.5	3.6	4.1	4.6	**4.9**	5.3	5.6	5.9
72	4.3	5.0	5.6	**6.1**	6.6	7.0	7.4	3.5	4.0	4.5	**4.8**	5.2	5.5	5.8
74	4.4	5.0	5.6	**6.1**	6.6	7.0	7.4	3.5	4.0	4.4	**4.7**	5.1	5.4	5.6
76	4.3	5.0	5.6	**6.1**	6.5	7.0	7.4	3.4	3.9	4.3	**4.6**	4.9	5.2	5.5
78	4.3	5.0	5.5	**6.1**	6.5	6.9	7.3	3.3	3.8	4.2	**4.5**	4.8	5.1	5.4
80	4.3	4.9	5.5	**6.0**	6.5	6.9	7.3	3.3	3.7	4.1	**4.4**	4.7	5.0	5.2
82	4.3	4.9	5.4	**5.9**	6.4	6.8	7.2	3.2	3.7	4.0	**4.3**	4.6	4.9	5.1
84	4.2	4.8	5.4	**5.9**	6.3	6.7	7.1	3.2	3.6	4.0	**4.3**	4.5	4.8	5.0
86	4.1	4.7	5.3	**5.8**	6.2	6.6	7.0	3.1	3.6	3.9	**4.2**	4.4	4.7	4.9
88	4.0	4.7	5.2	**5.7**	6.1	6.5	6.8	3.1	3.5	3.8	**4.1**	4.4	4.6	4.8
90	4.0	4.6	5.1	**5.5**	6.0	6.3	6.7	3.1	3.5	3.8	**4.1**	4.3	4.5	4.7
92	3.9	4.4	5.0	**5.4**	5.8	6.2	6.5	3.1	3.5	3.8	**4.0**	4.2	4.5	4.6
94	3.8	4.3	4.8	**5.3**	5.7	6.0	6.4	3.1	3.4	3.7	**4.0**	4.2	4.4	4.6
96	3.7	4.2	4.7	**5.1**	5.5	5.9	6.2	3.1	3.4	3.7	**3.9**	4.2	4.3	4.5
98	3.5	4.1	4.6	**5.0**	5.4	5.7	6.1	3.0	3.4	3.7	**3.9**	4.1	4.3	4.5
100	3.4	4.0	4.4	**4.8**	5.2	5.6	5.9	3.0	3.4	3.6	**3.9**	4.1	4.2	4.4

Abbreviation: SRT-WR_{peak}=peak work rate attained at the Steep Ramp Test

Supplementary Table 8. Fat-free mass- and sex-related reference centiles for absolute SRT-WR_{peak}.

Fat-free mass (kg)	SRT-WR_{peak} (W) Males							Females						
	P3	P10	P25	**P50**	P75	P90	P97	P3	P10	P25	**P50**	P75	P90	P97
20	91	103	112	**120**	127	134	139	103	110	119	**128**	139	151	164
22	99	112	122	**131**	139	146	153	110	118	128	**138**	149	162	176
24	109	123	134	**144**	153	161	169	118	128	138	**149**	161	174	188
26	119	134	147	**158**	168	177	186	127	138	149	**161**	174	187	202
28	130	146	160	**172**	184	194	204	138	149	161	**174**	188	202	218
30	142	159	174	**188**	201	213	224	149	162	175	**189**	204	219	235
32	156	174	190	**205**	220	233	246	162	176	190	**205**	221	237	253
34	169	189	206	**223**	239	254	268	175	190	206	**222**	239	255	273
36	183	204	223	**241**	258	275	291	189	205	222	**240**	257	275	293
38	198	219	239	**259**	278	296	314	203	221	239	**257**	276	295	314
40	212	234	255	**276**	297	317	337	216	235	255	**274**	294	315	335
42	227	250	272	**294**	316	338	360	228	249	270	**290**	312	333	354
44	243	266	289	**312**	336	360	383	239	261	283	**305**	327	349	371
46	258	282	306	**330**	355	380	406	247	271	294	**317**	340	363	386
48	273	297	322	**347**	373	400	428	253	278	303	**327**	351	375	398
50	287	312	337	**363**	390	418	447	258	285	311	**336**	360	384	407
52	300	325	351	**378**	406	435	465	261	290	318	**344**	368	392	415
54	312	338	364	**392**	420	450	481	263	295	324	**351**	376	399	422
56	323	349	376	**405**	434	464	496	265	299	330	**357**	382	406	428
58	334	360	388	**417**	447	478	509	267	304	335	**363**	389	412	433
60	344	371	400	**429**	459	490	522	268	308	341	**369**	395	418	439
62	353	382	411	**441**	471	502	534	269	312	346	**375**	401	424	445
64	363	392	422	**452**	483	514	546	269	316	352	**381**	407	430	450
66	372	402	433	**463**	494	525	556	270	320	357	**387**	413	436	456
68	381	412	443	**474**	505	535	566							
70	390	422	453	**484**	515	545	575							
72	399	432	463	**494**	525	555	584							
74	408	442	474	**505**	535	564	593							
76	418	451	483	**514**	544	573	601							
78	427	461	493	**524**	553	581	609							
80	436	470	503	**533**	562	590	616							

Abbreviation: SRT-WR_{peak}=peak work rate attained at the Steep Ramp Test

Supplementary Table 9. Fat-free mass- and sex-related reference centiles for SRT-WR$_{peak}$ normalized for body mass.

Fat-free mass	SRT-WR$_{peak}$ (W/kg)													
	Males							Females						
(kg)	P3	P10	P25	**P50**	P75	P90	P97	P3	P10	P25	**P50**	P75	P90	P97
20	3.3	4.0	4.5	**5.0**	5.3	5.7	6.0	3.4	3.9	4.5	**5.0**	5.6	6.2	6.8
22	3.3	4.0	4.5	**5.0**	5.4	5.7	6.1	3.4	3.9	4.5	**5.0**	5.5	6.1	6.7
24	3.4	4.0	4.6	**5.0**	5.4	5.8	6.1	3.5	3.9	4.4	**5.0**	5.5	6.0	6.6
26	3.4	4.1	4.6	**5.1**	5.5	5.9	6.2	3.5	4.0	4.5	**5.0**	5.5	6.0	6.5
28	3.4	4.1	4.7	**5.1**	5.6	5.9	6.3	3.5	4.0	4.5	**5.0**	5.5	6.0	6.5
30	3.5	4.2	4.7	**5.2**	5.7	6.1	6.4	3.6	4.0	4.5	**5.0**	5.5	6.0	6.5
32	3.6	4.3	4.8	**5.3**	5.8	6.2	6.6	3.6	4.1	4.6	**5.1**	5.5	6.0	6.5
34	3.7	4.4	4.9	**5.5**	5.9	6.4	6.8	3.7	4.2	4.7	**5.1**	5.6	6.0	6.5
36	3.8	4.5	5.0	**5.6**	6.1	6.5	6.9	3.8	4.3	4.7	**5.2**	5.6	6.0	6.5
38	3.9	4.6	5.1	**5.7**	6.2	6.7	7.1	3.8	4.3	4.8	**5.2**	5.7	6.1	6.5
40	4.0	4.6	5.2	**5.8**	6.3	6.8	7.2	3.9	4.4	4.8	**5.3**	5.7	6.1	6.4
42	4.1	4.7	5.3	**5.9**	6.4	6.9	7.4	3.9	4.4	4.9	**5.3**	5.7	6.1	6.4
44	4.1	4.8	5.4	**6.0**	6.5	7.0	7.5	3.9	4.4	4.9	**5.3**	5.7	6.0	6.4
46	4.2	4.9	5.5	**6.0**	6.5	7.0	7.5	3.9	4.4	4.8	**5.3**	5.6	6.0	6.3
48	4.3	4.9	5.5	**6.1**	6.6	7.1	7.6	3.8	4.3	4.8	**5.2**	5.6	5.9	6.3
50	4.3	4.9	5.5	**6.1**	6.6	7.1	7.6	3.7	4.3	4.7	**5.1**	5.5	5.8	6.2
52	4.3	5.0	5.5	**6.1**	6.6	7.1	7.5	3.6	4.2	4.6	**5.0**	5.4	5.7	6.0
54	4.3	5.0	5.5	**6.1**	6.6	7.0	7.5	3.5	4.0	4.5	**4.9**	5.3	5.6	5.9
56	4.3	5.0	5.5	**6.1**	6.5	7.0	7.4	3.3	3.9	4.4	**4.7**	5.1	5.4	5.7
58	4.3	5.0	5.5	**6.1**	6.5	7.0	7.4	3.2	3.7	4.2	**4.6**	4.9	5.2	5.5
60	4.3	5.0	5.5	**6.1**	6.5	7.0	7.4	3.0	3.6	4.0	**4.4**	4.7	5.0	5.3
62	4.3	5.0	5.6	**6.1**	6.5	7.0	7.3	2.9	3.4	3.8	**4.2**	4.5	4.7	5.0
64	4.3	5.0	5.6	**6.1**	6.5	6.9	7.3	2.7	3.2	3.6	**3.9**	4.2	4.5	4.7
66	4.3	5.0	5.6	**6.1**	6.5	6.9	7.3	2.5	3.0	3.4	**3.7**	4.0	4.3	4.5
68	4.3	5.0	5.6	**6.1**	6.5	6.9	7.3							
70	4.3	5.0	5.6	**6.1**	6.5	6.9	7.2							
72	4.3	5.1	5.6	**6.1**	6.5	6.9	7.2							
74	4.4	5.1	5.6	**6.1**	6.5	6.9	7.2							
76	4.4	5.1	5.7	**6.1**	6.5	6.9	7.2							
78	4.4	5.1	5.7	**6.1**	6.5	6.8	7.1							
80	4.5	5.2	5.7	**6.1**	6.5	6.8	7.1							

Abbreviation: SRT-WR$_{peak}$=peak work rate attained at the Steep Ramp Test

Supplementary Table 10. Fat-free mass- and sex-related reference centiles for SRT-WR_{peak} normalized for fat-free mass.

	SRT-WR_{peak} (W/kg FFM)														
Fat-free mass	Males								Females						
(kg)	P3	P10	P25	**P50**	P75	P90	P97		P3	P10	P25	**P50**	P75	P90	P97
20	4.3	4.9	5.4	**5.8**	6.2	6.5	6.8	4.3	4.8	5.2	5.7	**6.1**	6.7	7.3	8.0
22	4.4	5.0	5.5	**5.9**	6.3	6.6	6.9	4.4	4.8	5.2	5.7	**6.2**	6.7	7.3	7.9
24	4.5	5.1	5.5	**6.0**	6.4	6.7	7.0	4.5	4.9	5.3	5.7	**6.2**	6.7	7.3	7.9
26	4.6	5.1	5.6	**6.1**	6.5	6.8	7.2	4.6	4.9	5.3	5.8	**6.2**	6.8	7.3	7.9
28	4.7	5.2	5.7	**6.2**	6.6	7.0	7.3	4.7	4.9	5.4	5.8	**6.3**	6.8	7.3	7.9
30	4.8	5.3	5.8	**6.3**	6.7	7.1	7.5	4.8	5.0	5.4	5.9	**6.4**	6.9	7.4	7.9
32	4.9	5.4	5.9	**6.4**	6.9	7.3	7.7	4.9	5.1	5.5	6.0	**6.5**	7.0	7.5	8.0
34	5.0	5.5	6.1	**6.5**	7.0	7.5	7.9	5.0	5.1	5.6	6.1	**6.6**	7.1	7.6	8.1
36	5.1	5.6	6.2	**6.7**	7.2	7.6	8.1	5.1	5.2	5.7	6.2	**6.7**	7.2	7.7	8.2
38	5.2	5.7	6.3	**6.8**	7.3	7.8	8.3	5.2	5.3	5.8	6.3	**6.8**	7.2	7.7	8.3
40	5.3	5.8	6.4	**6.9**	7.4	7.9	8.4	5.3	5.4	5.9	6.3	**6.8**	7.3	7.8	8.3
42	5.4	5.9	6.5	**7.0**	7.5	8.1	8.6	5.4	5.4	5.9	6.4	**6.9**	7.4	7.9	8.4
44	5.5	6.0	6.6	**7.1**	7.6	8.2	8.7	5.5	5.4	5.9	6.4	**6.9**	7.4	7.9	8.4
46	5.6	6.1	6.6	**7.2**	7.7	8.3	8.8	5.6	5.4	5.9	6.4	**6.9**	7.4	7.9	8.4
48	5.7	6.2	6.7	**7.2**	7.8	8.3	8.9	5.7	5.3	5.8	6.3	**6.8**	7.3	7.8	8.3
50	5.8	6.2	6.7	**7.3**	7.8	8.4	9.0	5.8	5.2	5.7	6.2	**6.7**	7.2	7.7	8.2
52	5.8	6.3	6.8	**7.3**	7.8	8.4	9.0	5.8	5.0	5.6	6.1	**6.6**	7.1	7.6	8.0
54	5.8	6.3	6.8	**7.3**	7.8	8.4	8.9	5.8	4.9	5.5	6.0	**6.5**	7.0	7.4	7.8
56	5.8	6.3	6.7	**7.2**	7.8	8.3	8.9	5.8	4.7	5.4	5.9	**6.4**	6.9	7.3	7.7
58	5.8	6.2	6.7	**7.2**	7.7	8.2	8.8	5.8	4.6	5.2	5.8	**6.3**	6.7	7.1	7.5
60	5.7	6.2	6.7	**7.2**	7.7	8.2	8.7	5.7	4.5	5.1	5.7	**6.2**	6.6	7.0	7.3
62	5.7	6.2	6.6	**7.1**	7.6	8.1	8.6	5.7	4.3	5.0	5.6	**6.0**	6.5	6.8	7.2
64	5.7	6.1	6.6	**7.1**	7.6	8.0	8.5	5.7	4.1	4.9	5.5	**5.9**	6.3	6.7	7.0
66	5.6	6.1	6.6	**7.0**	7.5	8.0	8.4	5.6	4.0	4.8	5.3	**5.8**	6.2	6.5	6.8
68	5.6	6.1	6.5	**7.0**	7.4	7.9	8.3	5.6							
70	5.6	6.0	6.5	**6.9**	7.4	7.8	8.2	5.6							
72	5.5	6.0	6.4	**6.9**	7.3	7.7	8.1	5.5							
74	5.5	6.0	6.4	**6.8**	7.2	7.6	8.0	5.5							
76	5.5	5.9	6.3	**6.7**	7.1	7.5	7.9	5.5							
78	5.4	5.9	6.3	**6.7**	7.0	7.4	7.7	5.4							
80	5.4	5.8	6.2	**6.6**	6.9	7.3	7.6	5.4							

Abbreviations: FFM=fat-free mass; WR_{peak}=peak work rate.
